# Gender Differences in the Motivational Processing of Babies Are Determined by Their Facial Attractiveness

**DOI:** 10.1371/journal.pone.0006042

**Published:** 2009-06-24

**Authors:** Rinah Yamamoto, Dan Ariely, Won Chi, Daniel D. Langleben, Igor Elman

**Affiliations:** 1 Clinical Psychopathology Laboratory, McLean Hospital and Harvard Medical School, Boston, Massachusetts, United States of America; 2 Program in Media Arts and Sciences and Sloan School of Management, Massachusetts Institute of Technology, Cambridge, Massachusetts, United States of America; 3 Department of Psychiatry, University of Pennsylvania, Philadelphia, Pennsylvania, United States of America; L'université Pierre et Marie Curie, France

## Abstract

**Background:**

This study sought to determine how esthetic appearance of babies may affect their motivational processing by the adults.

**Methodology and Principal Findings:**

Healthy men and women were administered two laboratory-based tasks: a) key pressing to change the viewing time of normal-looking babies and of those with abnormal facial features (e.g., cleft palate, strabismus, skin disorders, Down's syndrome and fetal alcohol syndrome) and b) attractiveness ratings of these images. Exposure to the babies' images produced two different response patterns: for normal babies, there was a similar effort by the two groups to extend the visual processing with lower attractiveness ratings by men; for abnormal babies, women exerted greater effort to shorten the viewing time despite attractiveness ratings comparable to the men.

**Conclusions:**

These results indicate that gender differences in the motivational processing of babies include excessive (relative to the esthetic valuation) motivation to extend the viewing time of normal babies by men vs. shortening the exposure to the abnormal babies by women. Such gender-specific incentive sensitization phenomenon may reflect an evolutionary-derived need for diversion of limited resources to the nurturance of healthy offspring.

## Introduction

In men, heightened motivational drive for pursuit of heterosexual beauty was observed in the context of a validated computer key press task determining the viewing duration of beautiful female faces. Specifically, healthy men rated beautiful female faces as highly attractive as healthy women did for beautiful males, but expended greater effort (via the computer key press task) to increase the viewing times of these same faces [1]. We interpreted such disproportionate (relative to the valuational assessments) motivational drive to represent a “normative incentive sensitization,” a term reserved for motivational targets that are “wanted” more than could be explained by their hedonic properties, that is to say, “liking” [2].

A question that remained unanswered by our previous experimental design concerns an existence of the incentive sensitization phenomenon in women. If gender differences in social attachment are evolutionarily derived from conflicting motivations for maternal care vs. maximizing the number of fertilized women [3], pictures of babies (rather than of men) could be a sensitized motivational target for women [1]. To assess this possibility we modified our original task by substituting adult facial images with babies, while keeping all other task parameters unaltered.

Since motivation for viewing the images is not a unitary state characterized by only one pattern of behavior and emotions, we also assessed a potential influence of the perceived facial esthetics. Because the existing empirical data on specific characteristics conferring attractiveness features to a baby face are quite limited [4] with prior studies mostly focusing on Lorenz's “*Kindchenschema*” or babyishness [5], [6], [7], [8], [9] rather than on the attractiveness *per se*, we resorted to a categorical approach by including pictures of normal-looking babies and of those with abnormal facial features (e.g., cleft palate, strabismus, skin disorders, Down's syndrome and fetal alcohol syndrome). The validity of such choice is supported by a prospective survey of 1,450 children born with defects that revealed the decisive role played by esthetic appearance in motivation to care for children [10]. In that study, almost 70% of abandoned children carried a conspicuous appearance flaw that was neither life threatening nor did it affect intellectual development: only 7% of the abandoned children had a serious internal organ (e.g., heart and kidneys) disease. Additionally, the non-abandoned babies with an appearance flaw were commonly abused and isolated from their siblings by the caregivers [10].

Our above-mentioned key press/rating procedure may have a heuristic value for evaluation of potential impacts of facial esthetic features on motivational processing of babies. Thus, if perceived as rewarding, normal babies' images can be viewed longer than the abnormal ones, as determined by work in the units of the computer key press. On the other hand, pictures of abnormal babies can be evaluated as aversive using an objective marker, operant key-press behavior. Active avoidance of such aversive stimuli can be likewise rewarding and reinforcing [11], [12], [13] and thus serve as an additional (to normal babies) behavioral probe of motivational function. Incentive sensitization in these cases can be deduced from a heightened ratio of the key press effort to the attractiveness rating.

Weiss [10] did not methodically address gender differences in the attitudes toward children's appearance, and theoretical considerations on this score are not unambiguous. Therefore directional prediction on women's incentive sensitization (greater effort to view normal or not to view abnormal babies) was not sufficiently justified and the hypothesis was formulated in terms of gender-related differences in the motivational processing of babies' facial attractiveness.

## Materials and Methods

### Ethics Statement

All participants gave written informed consent to the McLean Hospital Institutional Review Board approved protocol after the procedure was fully explained.

### Participants

Participants were healthy men (n = 13, 4 parents) and women (n = 14, 2 parents), average age±SD: 38±14 for men and 34±11 for women [t(25) = 0.9, n.s.]. There were no significant differences in their ethnic breakdown, years of education or in their marital or parental status (p>0.2).

### Stimuli

The experimental paradigm was modeled after that of Aharon and colleagues [1], [14], [15]. Participants were presented with 80 images of infant faces; 50 images of normal baby faces and 30 images of abnormal baby faces, matched on sex and ethnicity (Caucasian, African-American, Asian, and Latin). Number of normal babies' images exceeded that of the abnormal ones to adjust for a potentially greater salience of negative than positive stimuli that was assumed to parallel greater salience attributed to losses over gains [16]. The facial abnormalities included strabismus, skin disorders, fetal alcohol syndrome, Down's syndrome and cleft palate. All images were culled from copy write free Internet resources (e.g., http://homepage.powerup.com/au/~cleftpal/photogallery.htm and http://www.kidsandbibs.com/photogallery/index.php).

The stimuli were standardized for size and equalization of the distances between standard facial landmarks (pupil to pupil distance 4 cm and temple to temple distance 9 cm) to ensure symmetry [17]. A black round frame (11.5 cm diameter), to allow only the face to be viewed, masked the images. The size of the image and the frame was 889×1097 dpi with RGB color. Adobe Photoshop 7.0 (Adobe) was used to create the masks and to ensure image consistency. The 80 images were presented in a random order using Authorware (Macromedia) on a Dell™ laptop computer with a 15-inch monitor. Participants were seated 16–18 inches from the monitor.

### Procedure

Two tasks were administered in separate runs: key press was followed by attractiveness rating of the images. Participants were informed that the overall duration of the key press task was fixed and independent of their actions, but they could control the amount of time they viewed each individual image. The default viewing time for an individual image was set at 4 seconds. The participant could adjust the viewing time for an image depending on the frequency of their key presses. Pressing the “z” or “m” keys could respectively increase or decrease the viewing time to 0 or 8 seconds. The “z” key presses were scored as positive, while the “m” key presses were scored as negative. The relationship between the key press effort and the viewing time is mathematically expressed as: NewTotalTime = OldTotalTime + (ExtremeTime – OldTotalTime)/K, where ExtremeTime is 0 and 8 seconds for the key presses respectively aimed to decrease or increase the viewing time; the scaling constant K is set at 40. This equation entails decreased efficacy of each successive key press with respect to changing the viewing time [14]. Such an exponential relationship between response and reinforcement rates is considered by some to be the superior strategy for the maintenance of operant behavior in both laboratory animals and in humans [18]. In the second task, participants rated the same images they had previously seen on a visual analog scale anchored by “not attractive at all” (0) and by “very attractive” (100).

### Statistical analyses

Data were analyzed using SPSS 13 for Mac OS X (SPSS Inc., Chicago, IL) T-tests for independent samples or χ^2^ statistics (when appropriate) were conducted to compare demographic variables. The net key press data and attractiveness rating were analyzed by means of Student's t-tests between the men and women groups for each of the two facial categories. Group data were summarized as mean±SD. All analyses were two-tailed and a *p* value <0.05 defined statistical significance.

## Results

**Figure 1:**
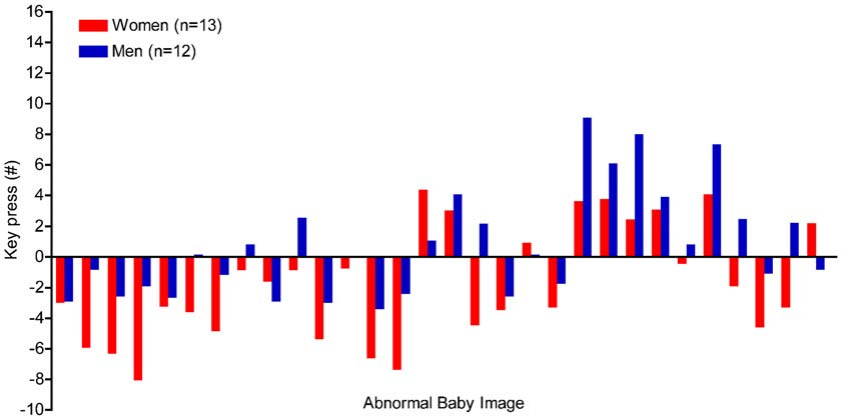
Performance on the key press task by men and women study participants. Data are presented as average key press number per image and are shown for images of abnormal babies.

Table 1 presents average key presses, ratings and key press/rating ratios for normal and abnormal facial images. Men expended similar effort to extend the viewing time of the normal babies faces but their attractiveness ratings for these images were significantly lower than in women. For abnormal babies, women provided similar attractiveness ratings to men, but their effort to avoid viewing the images exceeded that of men (Figure 1). Parallel to the key press results, women showed significantly shorter viewing times of abnormal babies as compared to men [3.6±0.5 sec vs. 4.0±0.5 sec, t(58) = 2.77, p = 0.007]; viewing times of normal babies were not significantly different between the groups [5.5±0.6 sec vs. 5.4±0.6 sec, t(98) = 0.17, p = 0.87].

**Table 1:** Performance on the key press task and facial attractiveness ratings by men and women study participants.

	Rating (mm)		Key press (#)		Key press/rating	
	Normal	Abnormal	Normal	Abnormal	Normal	Abnormal
Men	53.94 (5.36)	25.71 (9.24)	10.77 (4.47)	0.7 (3.5)	0.20 (0.07)	−0.01 (0.13)
Women	69.67 (9.13)	25.76 (12.73)	10.20 (3.84)	−1.75 (3.75)	0.14 (0.05)	−0.22 (0.51)
t	10.50	0.02	0.49	2.61	4.28	2.19
p	<0.001	0.99	0.69	0.01	<0.001	0.03

Data are presented as Mean (SD).

A subsequent analysis controlled for potentially confounding effects of baby face gender via analysis of covariance. Analyses of these data yielded essentially unchanged group effects: F(1,56) = 7.28, p = 0.009 for abnormal faces' key presses; F(1,96) = 0.49, p = 0.49 for normal faces' key presses; F(1,56) = 0.02, p = 0.90 for abnormal faces' ratings and F(1,96) = 108.79, p<0.001 for normal faces' ratings.

Absolute average numbers of key presses, regardless of whether scored positive or negative were not different between men and women [7.75±5.45 vs. 7.72±4.58, t(158) = 0.03, p = 0.97] indicating that group differences in the key presses for the abnormal facial category did not merely reflected a group difference in the general key press activity. Comparison of key press to ratings ratios was performed to provide an index of incentive sensitization. Absolute value of this ratio was elevated in men for normal babies and in women for abnormal babies. Finally, parents and non- parents performed similarly with regard to the key press and attractiveness rating on each of the normal and abnormal baby images.

## Discussion

We found similar motivational effort for viewing normal babies in both groups despite lower attractiveness ratings by the men. On the other hand, women rated abnormal babies' faces as unattractive as did men, but they expended more absolute effort to decrease the viewing times of these same faces. This group difference was not explained by the overall level of key-press activity and by gender of the baby face. The small number of participants renders our results preliminary however, pending replication in follow up studies.

The performance of work in order to continue viewing pictures of healthy babies is consistent with preclinical studies where rat pups served as a reinforcing stimulus in bar-pressing operant chamber procedures [19], [20]. This preference of laboratory animals was, however, narrowly restricted to mothers i.e., nulliparas avoided while postpartum animals were attracted to the pups [21], [22]. In humans, on the other hand, images of unfamiliar babies appear to be reinforcing in general, i.e., regardless of the gender and/or of parental status of the participants, as parents and non-parents of both genders activated brain motivational regions when exposed to such stimuli [9].

The present data extend our prior findings of increased effort to rating ratio exhibited by men with regard to attractive female faces, which we referred to as a gender-specific incentive sensitization [1]. Although there were methodological similarities between the latter [1] and current study (e.g., enrollment of healthy men and women as well as the use of an analogous key press/rating procedure), there were also important differences, including a novel pictorial stimulus and a decreased valuational assessment rather than an increased motivational effort displayed by men. Together, our current and previous results suggest that, in comparison to women, men may be more motivationally sensitized to procreation-related esthetic stimuli. An alternative explanation is that higher attractiveness ratings for normal babies could reflect societal acceptability demands still perceiving women as predominant caregivers for the young [23]. Resolution of the motivational vs. social origin interpretation of observed gender differences will require additional studies utilizing various types of esthetic stimuli.

Our results generalize motivational sensitization processes to women and suggest a different mechanism by which these processes may be mediated. Thus, in some domains, women may be predominately driven by negative reinforcement and/or avoidance leaning rather than by positive reinforcement mechanisms that may be more typical of men. This assumption may provide at least partial explanation for excessive reactivity to stress and other negative stimuli in women [24], [25], [26].

Studies of abandoned and neglected children firmly link their abnormal appearance to the maltreatment by the caregivers [27], [28], [29]. This may be to some extent because adults' are unconsciously motivated to care for infants with healthy facial features indicating fitness for survival and to exclude the least fit [30]. The abandonment and neglect data [27], [28], [29] along with our findings may thus challenge the commonly held view of unconditional maternal love and acceptance of the offspring [31]. If mother's love is not unconditional, what is the condition? The present results provide indirect support for Weiss' [10] idea that babies' esthetic appearance has a motivating influence on the adults' caretaking behavior. Clinical implications of our findings in terms of predicting potential for abuse and neglect of children with abnormal facial characteristics may transpire in cross-sectional and prospective clinical trials involving populations at risk. Further research is also needed to determine gender differences in the neural substrate underlying incentive sensitization processes and how it may be involved in psychopathologies characterized by gender-specific courses, such as schizophrenia, substance use disorders and major depression.

## References

[1] Levy B, Ariely D, Mazar N, Chi W, Lukas S (2008). Gender differences in the motivational processing of facial beauty.. Learning and Motivation.

[2] Berridge KC, Robinson TE (2003). Parsing reward.. Trends Neurosci.

[3] Darwin CR (1871). The descent of man, and selection in relation to sex.

[4] Stephan CW, Langlois JH (1984). Baby beautiful: adult attributions of infant competence as a function of infant attractiveness.. Child Development.

[5] Berman PW, Cooper P, Mansfield P, Shields S, Abplanalp J (1975). Sex differences in attraction to infants: when do they occur?. Sex Roles.

[6] Glocker ML, Langleben DD, Ruparel K, Loughead JW, Gur RC (2009). Baby schema in infant faces induces cuteness perception and motivation for caretaking in adults.. Ethology.

[7] Glocker ML, Langleben DD, Ruparel K, Loughead JW, Valdez JN (2009). Baby schema modulates the brain reward system in nulliparous women.. Proc Natl Acad Sci U S A.

[8] Brosch T, Sander D, Scherer KR (2007). That baby caught my eye attention capture by infant faces.. Emotion.

[9] Kringelbach ML, Lehtonen A, Squire S, Harvey AG, Craske MG (2008). A Specific and Rapid Neural Signature for Parental Instinct.. PLoS ONE.

[10] Weiss M (1994). Conditional love: parents' attitudes toward handicapped children.

[11] Kim H, Shimojo S, Doherty JP (2006). Is Avoiding an Aversive Outcome Rewarding? Neural Substrates of Avoidance Learning in the Human Brain.. PLoS Biology.

[12] Solomon RL, Corbit JD (1974). An opponent-process theory of motivation: I. Temporal dynamics of affect.. Psychological Review.

[13] Seymour B, O'Doherty JP, Koltzenburg M, Wiech K, Frackowiak R (2005). Opponent appetitive-aversive neural processes underlie predictive learning of pain relief.. Nature Neuroscience.

[14] Aharon I, Etcoff N, Ariely D, Chabris CF, O'Connor E (2001). Beautiful faces have variable reward value: fMRI and behavioral evidence.. Neuron.

[15] Elman I, Ariely D, Mazar N, Aharon I, Lasko NB (2005). Probing reward function in post-traumatic stress disorder with beautiful facial images.. Psychiatry Res.

[16] Redelmeier DA, Rozin P, Kahneman D (1993). Understanding patients' decisions. Cognitive and emotional perspectives.. Jama.

[17] Rhodes G (2006). The evolutionary psychology of facial beauty.. Annu Rev Psychol.

[18] Bradshaw CM, Szabadi E, Bevan P (1976). Behavior of humans in variable-interval schedules of reinforcement.. J Exp Anal Behav.

[19] Lee A, Clancy S, Fleming AS (2000). Mother rats bar-press for pups: effects of lesions of the mpoa and limbic sites on maternal behavior and operant responding for pup-reinforcement.. Behav Brain Res.

[20] Wilsoncroft WE (1969). Babies by bar-press: Maternal behavior in the rat.. Behavior Research Methods & Instrumentation.

[21] Fleming AS, Luebke C (1981). Timidity prevents the virgin female rat from being a good mother: Emotionality differences between nulliparous and parturient females.. Physiology & Behavior.

[22] Numan M (2007). Motivational systems and the neural circuitry of maternal behavior in the rat.. Dev Psychobiol.

[23] Feldman SS, Nash SC (1978). Interest in Babies during Young Adulthood.. Child Development.

[24] Karlsgodt KH, Lukas SE, Elman I (2003). Psychosocial stress and the duration of cocaine use in non-treatment seeking individuals with cocaine dependence.. Am J Drug Alcohol Abuse.

[25] Olff M, Langeland W, Draijer N, Gersons BP (2007). Gender differences in posttraumatic stress disorder.. Psychol Bull.

[26] Troisi A (2001). Gender differences in vulnerability to social stress: a Darwinian perspective.. Physiol Behav.

[27] Barden RC, Ford ME, Jensen AG, Rogers-Salyer M, Salyer KE (1989). Effects of Craniofacial Deformity in Infancy on the Quality of Mother-Infant Interactions.. Child Development.

[28] Hibbard RA, Desch LW (2007). Maltreatment of children with disabilities.. Pediatrics.

[29] Kurdahi Badr L, Abdallah B (2001). Physical attractiveness of premature infants affects outcome at discharge from the NICU.. Infant Behavior and Development.

[30] Lorenz K (1943). Die angeborenen Formen möglicher Erfahrung.. Zeitschrift für Tierpsychologie.

[31] Bartels A, Semir Z (2004). The neural correlates of maternal and romantic love.. NeuroImage.

